# Effects of custom-made textile insoles on plantar pressure distribution and lower limb EMG activity during turning

**DOI:** 10.1186/s13047-016-0154-5

**Published:** 2016-07-13

**Authors:** W. T. Lo, D. P. Wong, K. L. Yick, S. P. Ng, J. Yip

**Affiliations:** Institute of Textiles and Clothing, The Hong Kong Polytechnic University, Hung Hom, Hong Kong; Human Performance Laboratory, Technological and Higher Education Institute of Hong Kong, Hung Hom, Hong Kong; Sports Therapy Centre, Technological and Higher Education Institute of Hong Kong, Hung Hom, Hong Kong; Hong Kong Community College, The Hong Kong Polytechnic University, Hung Hom, Hong Kong

**Keywords:** Pivot, Textile-based insoles, Plantar loading, Electromyography, Turning phases

## Abstract

**Background:**

Turning during locomotion involves considerable changes of the body’s center of mass and reduced stability, as well as lower limb kinematics and kinetics. However, many previous studies have been carried out to evaluate the effectiveness and applications of orthotic insoles as well as different types of orthotic materials in various clinical symptoms, which are focused primarily on straight line walking. Hence, the influence of custom-made insoles with the use of advanced three-dimensional spacer fabrics on biomechanics parameters in terms of plantar pressure distribution and lower limb electromyography during turning movement was studied.

**Methods:**

Twelve subjects performed 180-degree turning at a speed 3.07-3.74 km/h for five successful trials under 3 insoles conditions: wearing traditional ethylene vinyl acetate insoles and two different spacer-fabricated insoles, with the plantar pressure and lower limb muscle activity collected simultaneously. Turning movement was broken down into 3 phases for analysis: Turning initiation, turn around and turn termination.

**Results:**

There was a statistically significance difference in plantar pressure between the traditional insoles and the insoles made of a spacer fabric as the top layer (*p* < 0.05). Compared to the traditional insoles, insoles made of a spacer fabric reduced the peak pressure (>12 %) and pressure–time integral (>13 %) in toes, metatarsal head 1 and metatarsal heads 2–3 at turning initiation; (>15 %) and (>17 %) in medial midfoot and medial heel at turn around. Insoles with spacer fabrics on the top and middle layer reduced both pressure parameters (>18 %) in toes and MTH 1 at turn termination. In terms of muscle activities, insoles with two-layer spacer fabrics could lower maximum muscle activities of vastus lateralis (>16 %; *p* < 0.05) at turn around.

**Conclusions:**

Insoles with different fabrications could offer various pressure offloading patterns across the plantar and muscle activity changes while turning. Insoles with a spacer fabric on the top tend to reduce plantar pressure loading at different regions during turn initiation and turn around phases, while two-layer spacer-fabricated insoles may contribute to reduced vastus lateralis muscle activation during turn around. More importantly, this study provides a new dimension in the potential use of the textile-fabricated insoles which may widen the range of insole materials selection in the design and development of insoles so as to enhance the effectiveness of orthotic treatment.

## Background

Turning is a common occurrence, accounting for approximately 35-45 % of all steps in activities of daily living [1–3]. It is a complex process that consists of decelerating the forward motion, rotating the body, and stepping out toward a new direction. Various modulations associated with the control of lower limb muscles are required according to the phase of the movement [4–7]. Straight line walking requires equal forces imparted to the body from both limbs, while turning involves limb kinetic asymmetry, that is, the inside limb must differ from the outside limb during turning [2]. It requires increased ankle push-off force on the outside limb to push the center of mass in the direction of the turn and to rotate the trunk towards the turning direction, and increased mediolateral ground reaction forces of the inside limb throughout the stance phase to propel the body in the desired direction of travel [2, 8].

Foot orthotic treatment is one of the primary means to handle various foot problems such as reducing the occurrence or recurrence of ulceration which is due to the excessive shearing together with abnormal levels of repeated pressure that occur within the foot leading to severe damage to soft tissue [9, 10]. Even though some previous studies have been carried out to evaluate the effectiveness and applications of orthotic insoles as well as different types of orthotic materials in various clinical symptoms [11–15], much clinical attention has been focused primarily on the foot loading characteristics and their effects on posture during gait at straight line walking [16–20], and thus little is known about the foot-footwear interface during turning. Specifically, very few studies have attempted to examine the implications of foot orthotic inventions on plantar pressure and muscle activation pattern so as to improve the turning function and safety of the wearers.

Custom-made orthotic insoles, which are designed to relieve and reduce plantar pressure over a wider surface area, have been widely used and highly promoted in the treatment of foot deformities and/or neuropathic conditions of the foot [21–24]. They are primarily constructed from foam rubber, cellular polymer and/or soft cushioning materials as shock absorbers, or load distributors that can minimise shock transmissions to the foot. The design, choice of foam materials used for fabrication, and fitting of the orthotic insoles affect the foot-insole Interface pressures, comfort of walking, and eventually, the efficacy of the foot orthotic treatment [14, 25–27]. With the rapid development of advanced textile materials, three-dimensional spacer fabrics are adopted for various medical purposes to resist pressure induced by body weight [28–31]. Inherent to the special sandwiched construction, spacer fabrics have been demonstrated to have satisfactory transversal compressibility, porosity and excellent planar elasticity that function well for shock absorption, cushioning and breathability [30, 32–34]. They are highly breathable and offer a moisture free environment which in turn reduces the chances of skin maceration and increases the level of comfort when compared to other materials such as foam, neoprene and composites [35]. In addition, spacer fabrics can be engineered in terms of yarn type and structure to provide a wide variety of mechanical and thermal properties in accordance with the specific requirements for different applications [35, 36]. The key properties of spacer fabrics, together with their light weight construction, support their use in the construction of orthotic insoles. These are proposed, for the first time in the present study to alter the plantar loading behaviors and muscle activation patterns during human movement.

To improve the quality of foot rehabilitation treatment, a thorough understanding of foot plantar loading behaviors and lower limb muscles activation patterns when foot orthoses are worn during the process of turning is imperative. The purpose of this article was to explore whether the novel textile materials could have a better performance in plantar pressure and muscle activity reduction during turning in healthy participants. Hence, the specific aim of the study was to examine the changes of plantar pressure distribution and lower limb muscle activity in response to custom-made textile insoles and traditional insoles during different phases of turning. It was hypothesized that 1) the overall distribution of plantar pressure and muscle activities associated with turning could be reduced, 2) the magnitude of pressure and muscle activities reductions vary at different turning phases by the use of textile insoles made of spacer fabrics.

## Methods

### Study design

In this within-subject repeated measures study, the participants wore three types of insoles made with different materials combinations. The plantar pressure distribution and muscle activity were measured during the wear trials.

### Participants

Twelve healthy subjects, 6 men and 6 women, who did not have any history of orthopedic or neurological conditions and were free of foot pain during the time of study participated in the study. Their average age was 23.0 (SD: 4.3); and body mass index (BMI) was 20.3 kg/m^2^ (SD: 2.6). Their foot sizes ranged from European 40 to 43 (male) and 37 to 40 (female). All participants signed a written consent in accordance with the ethics policy on human subjects as stipulated by the university before participation. The experimental protocol was approval by the Human Subjects Ethics Sub-committee of the Research Committee.

### Structure of custom-fabricated shoe insoles

**Fig. 1:**
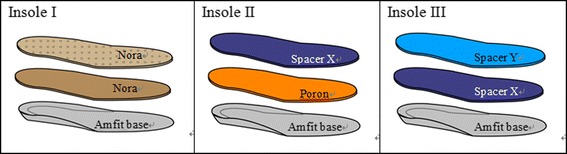
Insoles made of different combinations of materials

**Fig. 2:**
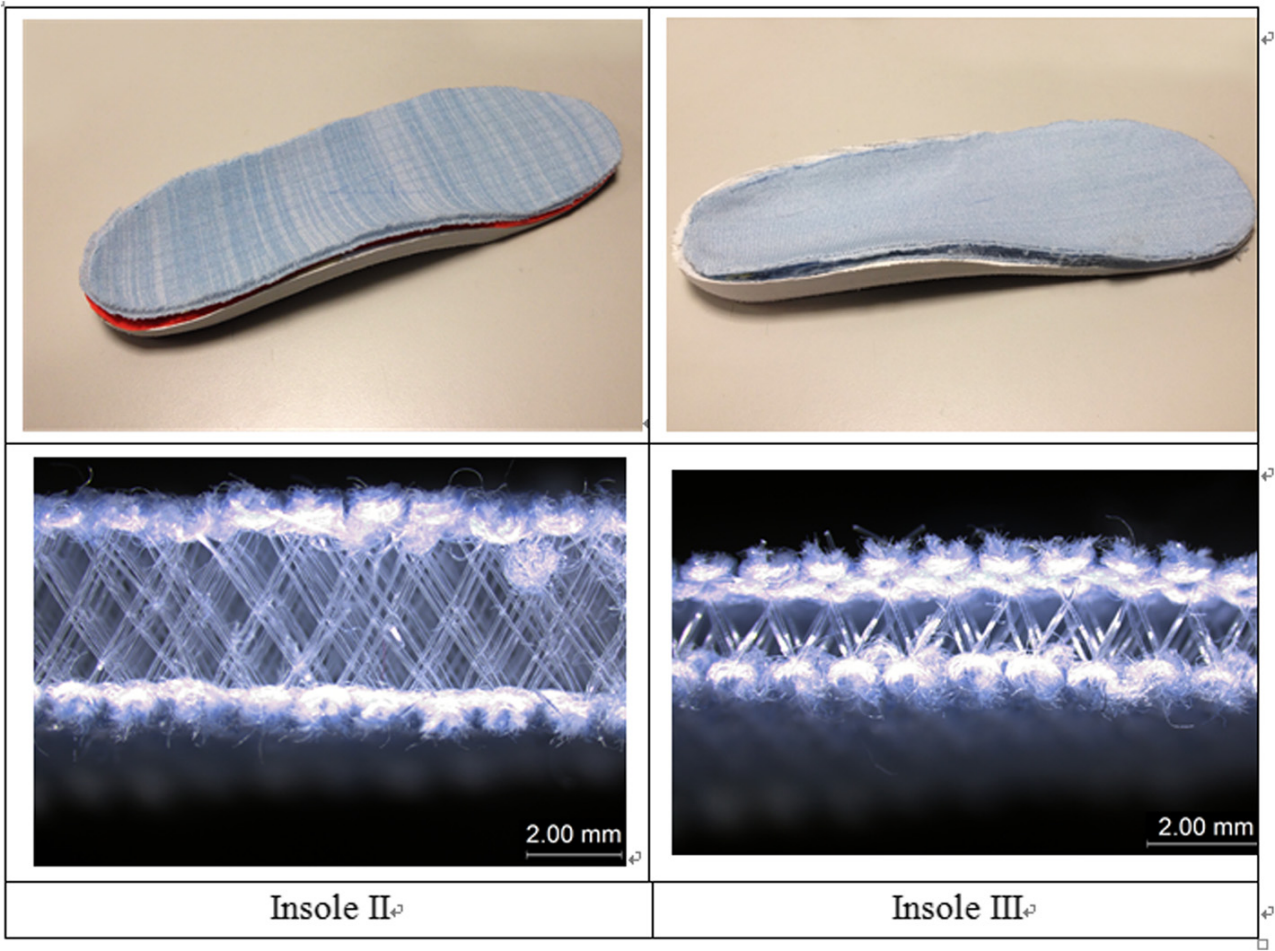
Prototypes of insole II (left) and insole III (right) and their corresponding top layers made of weft knitted 3D spacer fabrics

While the subjects were in an upright sitting position (half-weight bearing), the foot contour with the subtalar neutral position was captured by a contact digitizer of the Amfit® technology system (PN 10DDIGISYS-2, Amfit Incorporated, Vancouver, USA). The CAD/CAM mill connected to the digitizer receives the foot images and fabricates the 3D bottom layers of the insoles by a certified orthotist/prosthetist. Each participant was fitted with 3 pairs of custom-made multilayer insoles that were made with the same combinations of materials as shown in Table 1 and Fig. 1: the top and middle layers were glued onto the surface of the bottom layer. Two weft-knitted spacer fabrics X and Y (Fig. 2) have been developed by using a Stoll CMS 822 E7.2 computerized flat knitting machine. While the outer layers of the spacer fabrics were made from 150/48D polyester and nylon/spandex (70D/20D) yarn, the spacer layer was made of polyester monofilament with a diameter of 0.08 mm. The same fabrication method was used to construct the three-layer insoles for each subject. Their compression behavior and water vapour permeability are presented in Table 1.

**Table 1:** Summary of orthotic insole specifications

Insole	Top layer	Middle layer	Bottom layer	Description	Thickness of insole (mm)	Hardness (Shore A) ASTM D2240-05	Compression (kPa) ISO3386-1	Instant recovery rate (%) after compression ISO3386-1	Water vapor permeability (g/h · m^2^) ASTM E96
I	Nora® Lunairflex	Nora® Lunalastike	Amfit® Base	Top/Middle/Bottom: All are EVA	12	28	347.9	71.4	1.9
II	Spacer fabric X	Poron®	Amfit® Base	Top: Polyester Middle: polyurethane Bottom: EVA	14	18	46.8	48.1	5.0
III	Spacer fabric Y	Spacer fabric X	Amfit® Base	Top: Polyester Middle: Polyester Bottom: EVA	13	22	79.9	50.1	5.7

Note: EVA is ethyl vinyl acetate

### Test protocol

**Fig. 3:**
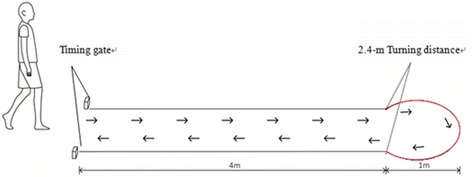
A walkway during wear trial

The data for each subject were collected on the same day. The subjects went through a 4-h acclimatising period of each insole wear (i.e. 12 h of insole wear in total for 3 insole conditions) for 3 to 4 days before the data acquisition. Previous studies have found that subjects, especially those with healthy foot conditions, probably feel uncomfortable for the contoured shape of the insoles and the overall comfort is likely influenced by arch comfort [37]. In this study, the tested insoles, are all custom-made with a contoured shape and arch support. The 4-h acclimatising period is used for neural adaptation in the sensory nerves acclimating the contour shape in response to standing, walking and turning motion. Each subject was invited to perform trials for all insole conditions on a level walkway (Fig. 3). Subjects were instructed to walk straight ahead for 4 meters, and then to perform a consistent turn in one direction of the semi-circular path with the dominant foot inside while turning during the trial, and to continue walking in the new direction for 4 additional meters. The pathway was designed to simulate the typical-sized turns often found in the local community and daily ambulation such as doorways and sidewalks pathways. Due to the limited space in the local living areas, the architectural features of these walkways usually require travel along with straight walking before and after the turn. The order of the 3 insoles (I, II, and III) was counterbalanced. The walking speed in all subsequent measurements was 3.49-3.96 km/h and 3.07-3.74 km/h of walking straight and turning respectively, and monitored by an automatic infrared timing gate (Brower Timing Systems, Utah, USA, 0.01 s precision) and maintained with a digital metronome since plantar pressure is highly dependent on speed, i.e., a higher speed is associated with higher plantar pressure [38–41]. Trials that had a walking speed outside the desired range were excluded and five valid trials per insole condition were eventually recorded for analysis. On the day of test, they were given sufficient practice walking trials to become accustomed to the next test condition and all equipment at the desired range of speed before data collection. A ten minutes rest with all equipment and shoes taken off was used in order to avoid the aggravation of pain during the tests and carried over to the next test condition.

#### Plantar pressure distribution measurement

**Fig. 4:**
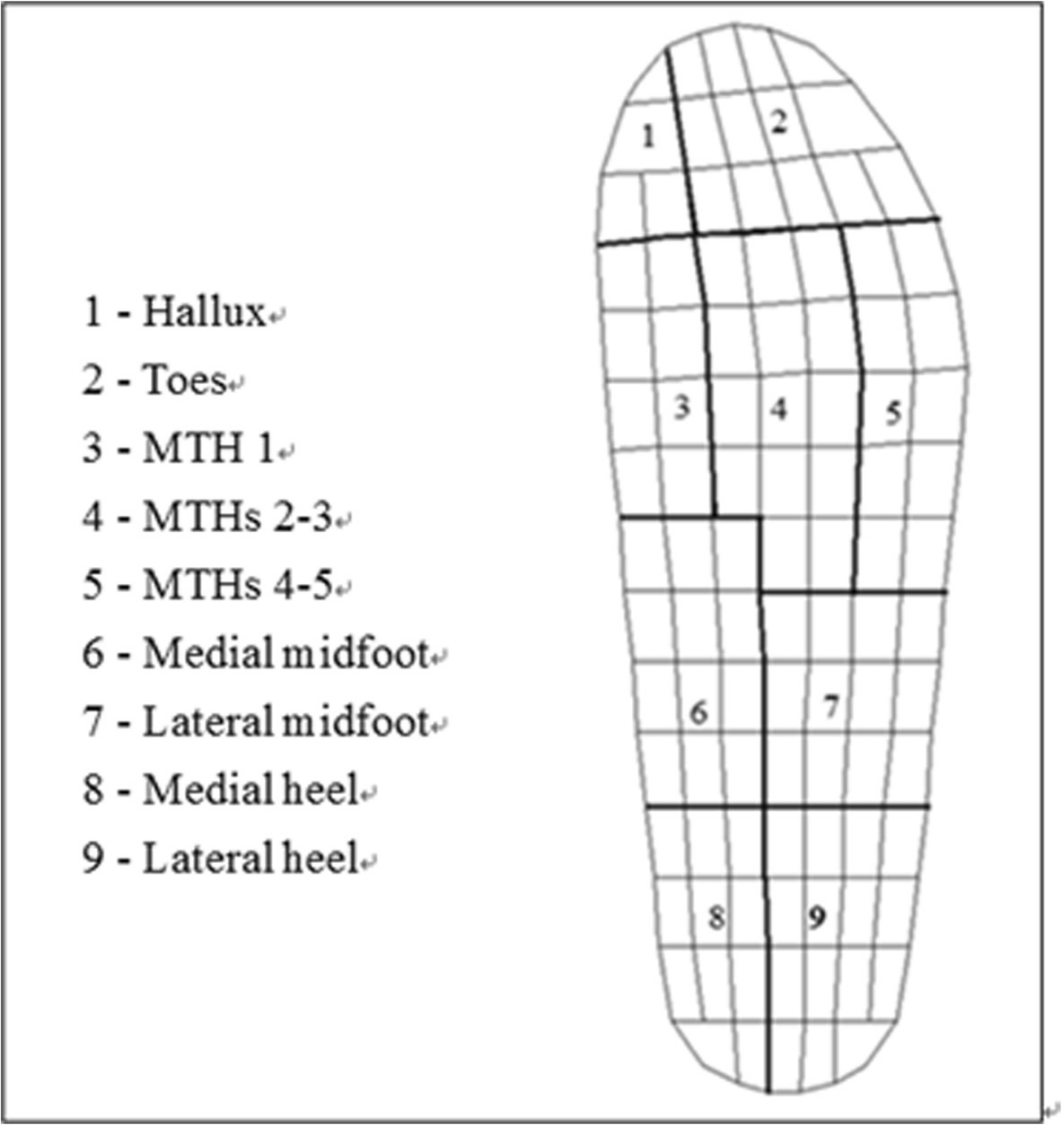
Nine subareas for each footprint

Pressure was measured at 160 Hz using the Pedar®-X in-shoe pressure measurement system (Novel GmbH, Munich, Germany) on the dominant foot. Each Pedar® insole sensor, which was 2 mm in thickness and consisted of sampling with 99 sensors, was fitted in accordance with foot size and placed inside each shoe between the foot and the insole. The participants wore the standard sports shoes, socks and insoles provided during data collection. The plantar pressure map was divided into nine subareas: hallux, toes, metatarsal head (MTH) 1, MTHs 2–3, MTHs 4–5, medial and lateral midfoot (MF) and medial and lateral heel by using the Novel Multimask software (Novel GmbH, Munich, Germany) as shown in Fig. 4 [42]. sEMG collection was synchronized with the video data recording by the sEMG system to monitor the whole trial, while the Pedar X-box with a top blue BT LED was sparkled to indicate an active Bluetooth connection between the Pedar-X box and the computer when the experiment is started. Video camera of the sEMG system was then used to capture and record sparkling light of the Pedar X-box from the onset. For each trial, four middle steps from walking straight and three steps including turn initiation, turn around and turn termination steps from turning were chosen and averaged. (All participants completed the entire turning with 3 steps while some participants had one more step either before or after turn around phases. Hence, three common steps were taken for analysis). The turn initiation and turn termination steps were determined based on the peak pressure (PP)/ pressure time integral (PTI) measured by using the Pedar system, in which the PP/PTI on the lateral side of the inner turning foot was noticeably deviated from straight-line walking gait. The turn around step was considered as the acute change of center of pressure line. The average of five successful trials was used for further analysis.

#### Lower limb muscle activity measurement

During the gait trials with each insole condition, surface electromyography (sEMG) data for the vastus lateralis, tibialis anterior and lateral gastrocnemius muscles were simultaneously collected with the plantar pressure distribution by using a 10-channel Datalog sEMG system at a sampling frequency of 2048 Hz (Thought Technology, USA). Bipolar Ag/AgCl surface electrode pairs were placed parallel to the alcohol cleaned and shaven surface of the skin overlying the corresponding muscle bellies. The electrodes were 10 mm in diameter and had 22 mm inter-electrode separation distance. Electrode placement followed the recommendations of Surface ElectroMyoGraphy for the Non-Invasive Assessment of Muscles (SENIAM) [43]. All sEMG signals were measured when participants were wearing the shoes and socks provided. Before the walking trial, maximum voluntary contractions (MVC) for each of the three muscle groups were acquired for 8 s by using manual resistance and repeated three times with five minutes rest in-between each time. The largest of the contraction for each muscle was defined as the MVC of the corresponding muscle. In each trial, the sEMG data of the steps from walking straight and turning which corresponded to the same steps used in the plantar pressure analysis were taken and averaged to ensure more reliable and relevant results [44]. The sEMG signals were full-wave rectified, and passed through a zero lag 4^th^ order Butterworth low-pass filter with a band-pass filtered at 10–500 Hz and stored for office analysis (Sacco, Akashi, and Hennig 2010). The sEMG data were analyzed by converting the data into root mean square values, which were calculated for a 400-ms window. The resultant maximum and mean amplitudes were averaged under each condition and normalized to the MVC of each subject which was expressed as a percentage. The average of five successful trials was then used for data analysis.

### Statistical analysis

All of the data are reported as mean (SD). The IBM SPSS software (version 16) was used for data analysis. Two-factor repeated measures analysis of variance (ANOVA) was performed to explore each measurement of the plantar pressure distribution, and muscle activity across the three insole conditions. There were two within-subject independent variables for each measurement: type of insole and subarea; and type of insole and muscle. In addition, one-factor repeated-measures ANOVA was used to further examine the difference between each dependent variable in each measurement among the three insole conditions. Bonferroni post hoc test was used for multiple pairwise comparisons when there was a demonstrated significant difference in both types of ANOVA.

## Results

### Plantar pressure distribution

Table 2 and Table 3 show the one-way repeated-measure ANOVA results of the PP and PTI respectively across all the plantar regions during turn initiation, turn around and turn termination among the three insole conditions. The change in PP and PTI patterns of three insoles at various subareas during different phases of turning is also presented.

**Table 2:** Peak pressure (kPa) in 9 plantar subareas during turn initiation, turn around and turn termination with the dominant foot inside while turning

	Hallux		Toes		MTH 1		MTHs 2-3		MTHs 4-5		Medial MF		lateral MF		Medial heel		Lateral heel	
Turn initiation																		
Insole I	175.9	(41.4)	143.1	(29.2)	139.4	(37.8)	125.0	(26.7)	101.4	(24.8)	60.2	(9.4)	88.5	(24.2)	116.7	(19.4)	134.5	(29.0)
Insole II	159.7	(40.2)	108.7	(23.7)	116.6	(29.4)	109.6	(22.2)	89.1	(26.3)	50.6	(8.4)	84.2	(20.8)	109.2	(28.6)	117.9	(28.3)
Insole III	158.4	(43.1)	110.8	(27.4)	129.7	(43.3)	121.4	(30.2)	90.7	(32.5)	57.8	(17.4)	80.2	(22.2)	118.0	(28.9)	123.5	(24.8)
Insole II % change	−9.2		−24.0		−16.3		−12.3		−12.2		−16.0		−4.8		−6.4		−12.4	
Insole III % change	−10.0		−22.6		−6.9		−2.9		−10.6		−4.0		−9.3		1.1		−8.2	
F-test value (η^2^) & significance			23.2 (0.68) *		3.5 (0.24) *		3.8 (0.26) *				3.7 (0.25) *							
Pairwise significance			I-II, I-III		I-II		I-II				I-II							
Turn around																		
Insole I	115.3	(41.7)	73.1	(29.8)	72.1	(32.3)	90.2	(29.9)	116.4	(37.0)	73.5	(15.2)	88.1	(32.5)	124.0	(23.7)	120.0	(28.0)
Insole II	106.5	(35.9)	68.3	(13.8)	68.1	(31.5)	82.5	(24.5)	112.2	(33.0)	61.9	(12.2)	84.3	(29.6)	100.3	(16.9)	100.3	(28.2)
Insole III	94.8	(19.6)	71.5	(19.0)	63.4	(30.9)	90.8	(31.9)	131.5	(60.1)	64.6	(13.8)	87.9	(29.6)	109.7	(20.4)	106.2	(23.3)
Insole II % change	−7.6		−6.6		−5.5		−8.5		−3.6		−15.8		−4.2		−19.1		−16.4	
Insole III % change	−17.7		−2.2		−12.1		0.6		12.9		−12.1		−0.2		−11.5		−11.5	
F-test value (η^2^) & significance											3.4 (0.24) *				8.6 (0.44) *			
Pairwise significance											I-II				I-II			
Turn termination																		
Insole I	149.3	(29.7)	146.7	(23.0)	122.6	(32.5)	137.2	(30.9)	118.2	(29.7)	56.2	(10.7)	106.0	(33.0)	119.2	(17.4)	125.7	(24.8)
Insole II	156.4	(42.0)	128.9	(23.1)	115.5	(33.9)	130.2	(30.4)	100.6	(27.8)	50.8	(11.5)	91.7	(26.3)	115.2	(22.2)	122.3	(29.7)
Insole III	121.8	(29.0)	120.3	(24.8)	99.7	(32.3)	143.8	(34.3)	116.1	(40.9)	52.6	(15.9)	105.6	(27.1)	125.1	(25.4)	124.4	(26.9)
Insole II % change	4.8		−12.2		−5.8		−5.1		−14.9		−9.6		−13.5		−3.4		−2.7	
Insole III % change	−18.4		−18.0		−18.7		4.9		−1.8		−6.4		−0.4		4.9		−1.0	
F-test value (η^2^) & significance	6.9 (0.39) *		8.8 (0.45) *		4.2 (0.28) *		6.6 (0.38) *											
Pairwise significance	I-III, II-III		I-II, I-III		I-III		II-III											

1) Mean (SD), 2) % change = % change in PP when compared with insole I; 3) *Significant difference of insoles in one-way ANOVA test at *p* < 0.05

**Table 3:** Pressure–time integral (kPa s) in 9 plantar subareas during turn initiation, turn around and turn termination with the dominant foot inside while turning

	Hallux		Toes		MTH 1		MTHs 2-3		MTHs 4-5		Medial MF		lateral MF		Medial heel		Lateral heel	
Turn initiation																		
Insole I	44.3	(12.7)	39.7	(9.0)	40.1	(11.1)	39.9	(8.7)	37.4	(8.0)	20.7	(3.8)	33.3	(10.7)	31.1	(6.1)	37.8	(19.4)
Insole II	39.7	(13.3)	30.3	(6.9)	32.2	(10.1)	34.5	(5.9)	34.4	(9.2)	17.8	(4.4)	30.2	(8.5)	28.6	(5.8)	33.4	(10.8)
Insole III	44.6	(21.2)	32.1	(8.2)	37.8	(14.8)	36.9	(10.2)	33.2	(9.3)	19.8	(5.2)	28.4	(6.8)	30.3	(7.8)	35.3	(15.7)
Insole II % change	−10.3		−23.7		−19.5		−13.6		−8.0		−14.1		−9.2		−8.0		−11.8	
Insole III % change	0.7		−19.0		−5.6		−7.5		−11.3		−4.1		−14.6		−2.7		−6.6	
F-test value (η^2^) & significance			18.8 (0.63) *		4.9 (0.31) *		5.6 (0.34) *											
Pairwise significance			I-II, I-III		I-II		I-II											
Turn around																		
Insole I	45.6	(14.4)	29.2	(11.9)	30.2	(11.3)	33.5	(14.4)	41.3	(11.9)	30.9	(5.2)	39.8	(17.2)	45.0	(11.1)	47.9	(20.1)
Insole II	36.8	(14.8)	25.5	(6.2)	26.5	(12.3)	29.8	(9.7)	34.3	(11.0)	25.6	(6.0)	36.5	(17.2)	34.0	(5.8)	36.8	(11.8)
Insole III	35.3	(5.6)	26.6	(7.2)	23.8	(10.7)	33.3	(12.1)	41.0	(14.9)	26.9	(8.1)	39.2	(15.3)	37.4	(10.0)	39.7	(16.9)
Insole II % change	−19.2		−12.6		−12.2		−11.0		−16.8		−17.2		−8.4		−24.5		−23.1	
Insole III % change	−22.6		−9.1		−21.3		−0.6		−0.5		−13.0		−1.7		−17.0		−16.9	
F-test value (η^2^) & significance	3.8 (0.26) *										3.7 (0.25) *				9.8 (0.47) *			
Pairwise significance	I-II										I-II				I-II, I-III			
Turn termination																		
Insole I	32.3	(11.8)	36.4	(9.1)	33.0	(12.2)	39.7	(13.4)	41.5	(11.6)	20.6	(6.6)	39.6	(14.9)	37.9	(9.5)	40.1	(20.0)
Insole II	33.6	(11.3)	30.0	(6.7)	28.1	(7.5)	34.7	(8.1)	33.7	(8.2)	19.2	(4.1)	32.3	(9.0)	33.8	(8.1)	37.6	(11.8)
Insole III	29.0	(13.2)	29.7	(6.0)	25.1	(9.9)	37.5	(13.1)	38.4	(12.4)	19.8	(6.9)	38.3	(11.0)	37.2	(11.0)	40.5	(16.4)
Insole II % change	4.1		−17.7		−14.7		−12.6		−18.9		−6.4		−18.4		−10.6		−6.3	
Insole III % change	−10.1		−18.5		−23.9		−5.6		−7.5		−3.5		−3.3		−1.7		1.1	
F-test value (η^2^) & significance			6.4 (0.37) *		5.5 (0.33) *													
Pairwise significance			I-III		I-III													

1) Mean (SD), 2) % change = % change in PTI when compared with insole I; 3) *Significant difference of insoles in one-way ANOVA test at *p* < 0.05

#### Peak pressure

The insoles and subareas have statistically significant effects on PP during turn initiation (F_2,22_ = 18.5, *p* < 0.001; F_8,88_ = 20.6, *p* < 0.001 respectively), and during turn termination (F_2,22_ = 6.8, *p* = 0.005; F_8,88_ = 16.4, *p* < 0.001 respectively). There is no interaction effect during turn initiation. The two-way interaction (insole-subareas) is only significant for the PP (*p* < 0.02) during turn termination. The post hoc tests indicate that when compared with insole I, there were statistically significant differences in PP during the turn initiation (120.5 vs. 105.1), turn around (97.0 vs. 87.2), and turn termination (120.1 vs. 112.4) in insole II. Insole II resulted in lower peak pressures compared to insole I during various turning phases. Reduction in PP was also found in insole III during turn initiation (120.5 vs. 110.1, *p* < 0.05) and turn termination (120.1 vs. 112.2, *p* < 0.05). No significant differences were shown with reference to turn around.

**Fig. 5:**
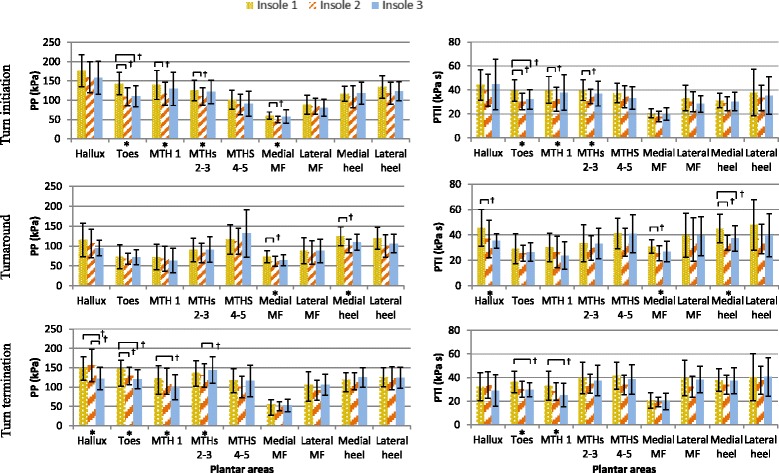
Mean PP and PTI distributions of all plantar subareas during turn initiation, turn around and turn termination of turning (standard deviation is marked on top of data bar). *Significant difference amongst insoles, †Pairwise significant between insoles

The results in Table 2 comparing different insole conditions indicate statistically significant differences in the toes, MTH 1 and MTHs 2–3 at turn initiation and turn termination (*p* < 0.05). Statistically significant pressure differences were also found in medial MF and medial heel at turn around. The Bonferroni pair-wise comparison showed significant pressure differences at toes (mean difference (SD): 34.4 (5.5), % change: 24.0 %, *p* < 0.001), MTH 1 (22.7 (8.4), 16.3 %, *p =* 0.01) and medial MF (9.6 (1), 16.0 %, *p* = 0.012) during turn initiation, and medial MF (11.6 (3.0), 15.8 %, *p* = 0.041) and medial heel (23.7 (6.8), 19.1 %, *p* = 0.005) during turn around between insole I and insole II. Similar trend of pressure reduction was also observed in Insole III at hallux (27.5 (0.7), 18.4 %, *p* = 0.011), toes (26.4 (1.8), 18.0 %, *p* = 0.007) and MTH 1 (23.0 (0.3), 18.7 %, *p* = 0.03) during turn termination when compared to Insole I. No significant differences were shown with reference to heel areas during turn initiation and termination, while no significant difference was found in forefoot areas during turn around. When comparing PP in different phases as shown in Fig. 5, regardless of insole types, hallux and MTH 1 shows the highest PP at turn initiation while MTHs 2–3, lateral MF and lateral heel display the highest PP at turn termination. In view of turn around, forefoot (except for MTHs 4–5) and hindfoot are found to have the lowest PP whereas MTHs 4–5 and medial MF are found to have the highest PP.

#### Pressure–time integral

Analysis of the PTI yielded similar results, whereby the insoles and subareas have statistically significant effects on the PTI during turn initiation (F_2,22_ = 8.0, *p* = 0.002; F_8,88_ = 7.8, *p* < 0.001 respectively), turn around (F_2,22_ = 7.5, *p* = 0.011; F_8,88_ = 5.3, *p* = 0.003 respectively) and turn termination (F_2,22_ = 4.4, *p* = 0.025; F_8,88_ = 7.4, *p* < 0.001 respectively). Nevertheless, none of the two-way interaction (insole-subareas) is significant. The post hoc tests indicate that insole II reduced PTI at turn initiation (36.0 vs. 31.2*, p* < 0.05) and turn around (38.2 vs. 31.8*, p* < 0.05) compared with insole I.

With reference to Table 3, the result reveals statistically significant differences in the toes and MTH 1 at turn initiation and turn termination (*p* < 0.05) amongst different insole conditions. Significant differences were also found in hallux, medial MF and medial heel at turn around. In the pairwise comparison, significant PTI differences at toes (9.4 (2.0), 23.7 %, *p* = 0.001), MTH 1 (7.8 (1.0), 19.5 %, *p* = 0.007) during turn initiation, and hallux (8.8 (0.4), 19.2 %, *p* = 0.02), medial MF (5.3 (0.9), 17.2 %, *p* = 0.025) and medial heel (11.0 (5.3), 24.5 %, *p* = 0.007) during turn around between insole I and insole II. Similar trend of PTI reduction was also observed in insole III at toes (9.4 (2.0), 18.5 %, *p* = 0.024) and MTH 1 (7.9 (2.4), 23.9 %, *p* = 0.013)) during turn termination when compared to insole I. No significant differences were shown with reference to midfoot and heel areas during turn initiation and termination.

### Lower limb electromyography

Table 4 presents the one-way repeated-measure ANOVA result of the maximum and mean muscle activities across all the muscles during turn initiation, turn around and turn termination among the three insole conditions. The change in muscle activity patterns of three insoles in muscle groups during different phases of turning is also presented.

**Table 4:** Muscle activity (normalized as% MVC) of vastus lateralis, tibialis anterior and lateral gastrocnemius during turn initiation, turn around and turn termination with the dominant foot inside while turning

	Maximum						Mean					
	Vastus lateralis		Tibialis anterior		Lateral gastrocnemius		Vastus lateralis		Tibialis anterior		Lateral gastrocnemius	
Turn initiation												
Insole I	18.4	(5.8)	21.6	(5.5)	21.9	(12.7)	7.5	(3.1)	10.9	(3.1)	8.3	(3.3)
Insole II	16.9	(5.7)	22.3	(6.3)	20.1	(7.5)	7.2	(2.7)	11.5	(3.6)	8.3	(3.2)
Insole III	17.7	(6.1)	20.7	(5.0)	21.7	(11.6)	7.2	(3.0)	10.5	(2.7)	8.3	(3.5)
Insole II % change	−8.5		2.9		−8.2		−3.7		5.8		0.7	
Insole III % change	−4.0		−4.2		−0.8		−4.0		−3.4		−0.2	
Turn around												
Insole I	22.6	(6.2)	20.4	(6.1)	25.1	(7.9)	9.1	(3.0)	11.8	(4.0)	9.5	(3.4)
Insole II	19.5	(6.5)	22.6	(7.9)	24.4	(6.8)	8.1	(2.4)	12.8	(5.0)	9.3	(3.8)
Insole III	18.8	(6.1)	21.3	(7.3)	25.6	(5.7)	7.9	(2.9)	11.5	(3.9)	10.5	(2.5)
Insole II % change	−13.6		10.5		−2.6		−11.8		8.3		−2.3	
Insole III % change	−16.9		4.3		2.1		−13.4		−3.1		9.8	
F-test value (η^2^) & significance	*											
Pairwise significance	I-III											
Turn termination												
Insole I	21.9	(6.5)	27.0	(9.7)	32.7	(15.8)	8.6	(3.2)	13.0	(4.7)	10.6	(3.7)
Insole II	18.7	(7.0)	24.0	(6.2)	29.5	(12.6)	7.7	(3.0)	12.1	(3.6)	10.4	(3.6)
Insole III	19.2	(6.0)	25.4	(9.4)	31.2	(17.5)	7.7	(3.0)	12.5	(4.5)	10.2	(4.2)
Insole II % change	−14.6		−11.0		−9.8		−9.9		−7.5		−1.6	
Insole III % change	−12.4		−5.9		−4.6		−9.8		−4.3		−3.4	

1) Mean (SD), 2) % change = % change in maximum/ mean muscle activity when compared with insole I; 3)*Significant difference of insoles in one-way ANOVA test at *p* < 0.05

#### Maximum muscle activity

**Fig. 6:**
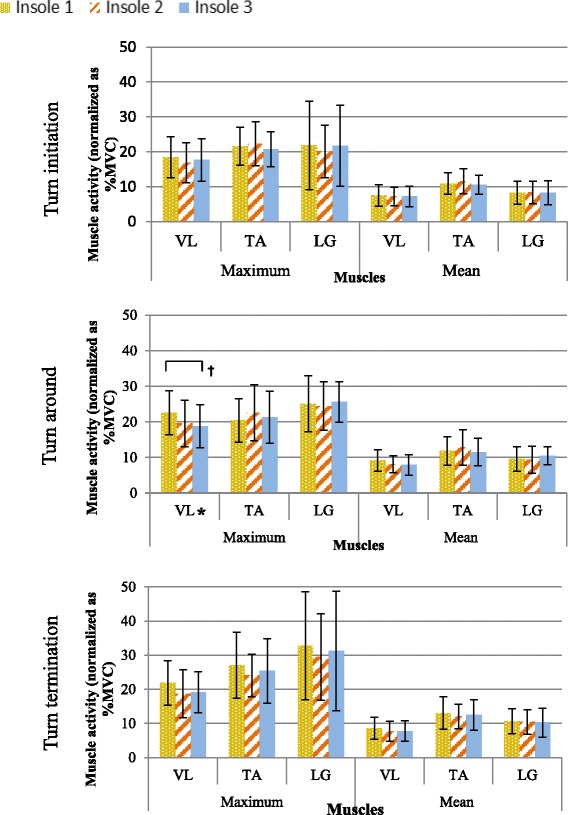
Mean muscle activity of all muscle groups during turn initiation, turn around and turn termination of turning (standard deviation is marked on top of data bar).*Significant difference amongst insoles, †Pairwise significant between insoles

The main effect of the muscle type (F_2,22_ = 4.9, *p* = 0.017) is only significant for the maximum muscle activity during turn termination. The two-way interaction (insole-muscle) or pair-wise comparison of insoles is not significant (*p* > 0.05). The results in Table 4 indicate that a significant difference (*p* < 0.05) is detected only in the vastus lateralis muscle across insoles during turn around, where insole III reduced the maximum muscle activity (3.8 (0.1), 16.9 %, *p* = 0.044) compared to insole I in the pairwise comparison. No significant differences were found in any muscle group amongst insole conditions during turn initiation and termination. Besides, when comparing maximum muscle activity in different phases as shown in Fig. 6, regardless of insole types, the muscle activity of all muscle groups at turn termination is higher than that of turn initiation, whilst vastus lateralis muscle activity is found to be the highest at turn around.

#### Mean muscle activity

The main effect of the insole type is significant for the mean muscle activity during turn initiation (F_2,22_ = 4.5, *p* = 0.024), turn around (F_2,22_ = 3.8, *p* = 0.037) and turn termination (F_2,22_ = 4.3, *p* = 0.026) but the two-way interaction (insole-muscle) is not significant (*p* > 0.05). The post hoc tests indicate that reduction in the mean muscle activity in insole I during turn initiation (7.3 vs. 11.0*, p* < 0.05), turn around (8.4 vs. 12.1*, p* < 0.05), and turn termination (8.0 vs. 12.5*, p* < 0.05) when compared to insole II. Results shown in Table 4 indicate that no significant difference is found in each muscle group across insoles or identified by the pair-wise comparisons.

## Discussion

While much of existent research has focused on walking straight or running associated with interventions or injury prevention, the present study highlights the importance of evaluating the biomechanical effects at different phases of turning. The purpose of this study was to investigate the effect of custom-made insoles for rehabilitation and the choice of material on plantar pressure and lower limb muscle activity during turn initiation, turn around and turn termination. The results support the hypothesis that the use of textile insoles made of spacer fabrics are better than traditional EVA insoles in reducing plantar pressure and lower limb muscle activity during turning. Wearing spacer-fabricated insoles also exhibited different reduction patterns of pressure and muscle activities during turn initiation, turn around and turn termination.

It is evident that the body center of mass is reoriented toward the inner foot during turning which leads to increase in plantar loading of the corresponding foot [4]. Such redistribution results in limb asymmetries which allow the body to shift the body center of mass towards the inner limb [45], thus inducing changes in the magnitude of the PP and PTI. In view of the different phases while turning as shown in Fig. 5, regardless of insole types, the change in PP pattern is different at various subareas. It is observed that there is a notable increase in the PP at hallux and MTH 1 when the turning is initiated and at MTHs 2–3 and lateral side of the foot when the turning is terminated by the inner leg to adjust gait trajectory back and forth between the straight-line and special designed turning pathway. There is obvious plantar pressure redistribution during the turn around phase to support the weight and maintain body stability in order to make a U-turn. Plantar pressures were observed to be reduced at forefoot (MTH 1, 2 and 3) and hindfoot and increased at MTHs 4–5 and medial midfoot.

In comparison to the traditional insole (insole I), textile-fabricated insoles could reduce pressure loading in various plantar subareas during different phases of the turning. In this study, when the insole consists of one layer of textile material (i.e. insole II), the PP and PTI are significantly reduced mostly in forefoot regions such as Toes, MTH 1 and MTHs 2–3 at turning initiation and a significant reduction in the medial side of midfoot and heel are found at turn around. In regard to the insole composing of two-layer textile materials (i.e. insole III), the pressure loading is significantly reduced in toes and MTH 1 during turn termination. This may be attributed to the soft sandwiched structure of spacer fabrics that spacer fabrics are used as the top layer of insoles II and III with direct contact with the foot for better resilience and recovery performance [32, 34], thus enhancing the overall foot-insole accommodation. In this case, insole II tends to have a more pronounced pressure reduction effect. Insole II, which is made of spacer fabric X and Poron® as the top accommodation layer and middle cushioning layer respectively, might effectively enhance pressure relief; whereas insole III, which is made of spacer fabric Y with a lower resilience to compressibility because of its low pile height and interlacing density structure, might show a comparatively lower pressure reduction performance than insole II [31, 46]. The findings suggest that spacer fabrics could have a good potential for insole fabrication. The physical properties of yarn and the change of interaction between the inner and outer layers could influence the structure and mechanism of spacer fabrics, which in turn could offer various orthotic performances. As spacer fabrics could be customized and engineered in terms of yarn type and structure as well as moulded for optimal fit, the application of spacer-fabricated insoles could further create a compression-resistant and climate-controlling zone inside the shoe.

In this study, the redistribution of the body center of mass may also induce the turn-related sensory feedback which leads to activate more muscles around the knee of the inner limb during the stance phase to enable body support and maintain whole body stability while turning [47–50]. For various muscle activations at different phases while turning as shown in Fig. 6, regardless of insole types, the muscle activation of all studied muscle groups is notably higher at turn termination than that of the turn initiation. The inner leg required increased muscle activity at turn termination may be necessary to generate supportive action and maintain whole body stability so as to readjust the gait from the turn around phase to the straight path. That may also explain why the demand of vastus lateralis maximum muscle activity is increased during turn around as more muscles are activated around the knee which may enable better body support during the change in direction in order to make a U turn.

With respect to the noticeable decrease in the maximum and mean muscle activity of almost all of the muscle groups after the use of textile insoles during different phases of turning, the reason may be related to the material features that make up the insoles. For instance, the viscoelastic and shock-absorbing properties of Poron® together with the transversal compressible spacer fabric with a high pile height in insole II could possibly reduce the frequency of the overuse of the foot [51]. This material combination may also provide more medio-lateral stability and better reduction in peak inversion for the foot, thus leading to less demand on the anti-pronatory muscles compared to the traditional insole I which is the hardest and stiffest that may increase postural preparation or muscle tension against a disturbance [52]. Nevertheless, tibialis anterior was modestly increased in the maximum and mean muscle contraction after wearing the insole II at turn initiation and turn around when compared to the control insole. The reason has not yet been identified from the present study, so further studies will be needed to clarify this issue.

Most previous studies have been conducted on investigating the effects of different orthotic insoles on plantar pressure distribution and muscular activities during straight line walking. The major novelty of our work is focusing on turning which had been further differentiated into turn initiation, turn around and turn termination. Related studies have reported that plantar peak pressure is redistributed across the plantar with the insertion of contoured shape insoles when compared to no insole condition. In Redmond’s study, custom made insoles with EVA insertion at heel posts could reduce peak pressure 12.7 % and 17.2 % at heel area when compared to prefabricated insole and control shoe conditions respectively [53]. Hinza indicated that wearing EVA insoles with anatomically shape could reduce the average pressure values at MTHs 2–3 by 9.7 % to 11.4 % as compared to the conventional insoles [54]. In the present study, the textile-fabricated insoles comprising of the sandwich feature of spacer fabric could reduce the pressure loading at various regions (0.6-12.6 % at MTHs 2–3 and 1.7-24.5 % at heel area) during different phases of turning when compared to insole I was made of EVA. The findings here could provide potential solutions to modify insole materials in future studies so as to optimize orthotic effects using biomechanical analyses.

It is noted that there are some limitations in this study. The sample size is relatively small and therefore might have limited the generalizability of the results. The current turning task was merely a single subset of turning gait, which may not be representative of all types of turns that occur during daily ambulation and of all human turnings. More rigorous and various types of turning in daily tasks would be necessary for further studies. Also, it is recognized that only the immediate effect of the insole was tested. Further research is required to evaluate the long term effects of the materials on pressure loading and to investigate the durability of the materials when exposed to prolonged wear and tear. As footwear comfort is emerging as an important research field, further work is necessary to identify the determining factors. It is recognized that the use of young healthy adults may be responsible for the lack of differentiation between the effects of the material on plantar and muscle response because they may have been able to modify their gait or plantar loading to prevent any foot areas from excessive stress. Nonetheless, the study provides preliminary evidence that supports the use of spacer fabrics in designing orthotic insoles, thereby providing a basis for future studies to highlight the high flexibility of spacer with its significant thermal features in further developments.

## Conclusions

Turning is one of the major motions and makes up a large portion of steps taken during activities and therefore should be taken account of during lower limb kinematic and kinetic investigations of different insole designs. The present study has provided a novel approach on the use of spacer fabricated insoles, which not only reduces/redistributes plantar pressure, but also lowers muscle activity of the lower limbs during various phases of turning. The wide availability, versatility and cost effectiveness of knitted spacer fabrics and/or advanced textile materials also allow practitioners to widen their selection of insole materials in the design and development of orthotic insoles. It is suggested that a spacer fabric is used as the top layer while a soft and cushioning material is used as the middle layer for orthotic insoles, and that different pile heights and thicknesses be used for patients with different needs and support requirements.

## Abbreviations

EVA, ethylene vinyl acetate; MF, midfoot; MTH, Metatarsal head; MVC, maximum voluntary contractions; PP, peak pressure; PTI, pressure time integral, sEMG, surface electromyography
